# Socio-Economic Status and Reproduction among Adults Born with an Oral Cleft: A Population-Based Cohort Study in Norway

**DOI:** 10.1371/journal.pone.0162196

**Published:** 2016-09-15

**Authors:** Erik Berg, Åse Sivertsen, Anja Maria Steinsland Ariansen, Charles Filip, Halvard A. Vindenes, Kristin B. Feragen, Dag Moster, Rolv Terje Lie, Øystein A. Haaland

**Affiliations:** 1 Department of Global Public Health and Primary Care, University of Bergen, Bergen, Norway; 2 Department of Plastic Surgery, Haukeland University Hospital, Bergen, Norway; 3 Norwegian Quality Registry of Cleft Lip and Palate, Bergen, Norway; 4 Department of Plastic and Reconstructive Surgery, Rikshospitalet, Oslo University Hospital, Oslo, Norway; 5 Centre for Rare Disorders, Oslo University Hospital, Oslo, Norway; 6 Norwegian Institute of Public Health, Bergen, Norway; 7 Department of Pediatrics, Haukeland University Hospital, Bergen, Norway; Medical University of South Carolina, UNITED STATES

## Abstract

**Background:**

It has been reported that people born with orofacial clefts do worse in life than their peers regarding a range of social markers, such as academic achievement and reproduction. We have compared otherwise healthy individuals with and without clefts, to investigate if these differences are due to the cleft or other background factors.

**Materials and Methods:**

In a retrospective national cohort study, based on compulsory registers with data collected prospectively, we included everybody born in Norway between 1967 and 1992 (1490279 individuals, 2584 with clefts). This cohort was followed until the year 2010, when the youngest individuals were 18 years old. In order to ensure that the individuals were not affected by unknown syndromes or diseases, we excluded all individuals with any chronic medical condition, or who had other birth defects than clefts, hydroceles and dislocated hips. Individuals with oral clefts who were included in the study are said to have isolated clefts.

**Results:**

Isolated cleft patients are similar to the general population regarding education, income and social class. Isolated cleft patients have lower fertility than the background population, but considering only married couples this difference in fertility disappeared.

**Conclusions:**

An oral cleft did not appear to affect future socioeconomic status or chances of becoming a parent for children born in Norway. An exception was males with cleft lip and palate, but differences were small.

## Introduction

Oral cleft is a common category of congenital anomalies. Worldwide the prevalence is about 1.7 per 1000 live births [1]. There are three major sub-groups with different etiology, severity and treatment: Cleft lip only (CLO), the more severe cleft lip with cleft palate (CLP), and cleft palate only (CPO) [1]. Early and multidisciplinary treatment is important [2]. Although clefts can be repaired surgically, children born with clefts may experience difficulties. They may have problems with breast feeding [3], speech and language development may be delayed [4], and hearing may be impaired [5], which may affect education and social integration during childhood. Treatment is extensive and time consuming, and teasing or lack of satisfaction with facial appearance may have a negative effect on self-esteem [6]. More generally, the presence of a cleft at birth is suspected to adversely affect quality of life in a number of areas, like health and mental health [7, 8], well-being and social life [9]. However, there is limited information on how an oral cleft affects the ability of otherwise healthy adolescents to cope with adult life, and previous studies are inconsistent [10].

Clefts are associated with other disorders, like genetic syndromes and developmental delays [11]. Clinical experience suggests that such disorders may be hard to observe directly, but may be seen indirectly through the presence of other medical problems. Congenital anomalies in addition to the cleft are commonly used as a crude indicator of underlying disorders. Clefts without additional congenital anomalies are referred to as isolated. Some individuals with apparently isolated clefts experience cognitive difficulties later in life [12]. Even if a medical diagnosis is not received until adolescence, this still suggests that the cleft was not really isolated [13]. A study that attempts to predict the consequences in adult life of an isolated oral cleft and its treatment should not include individuals with such diagnoses.

In order to study socio-economic status and reproduction among adults who were born with an isolated oral cleft, clinical files of all cleft cases treated in Norway from 1967 to 1992 were linked with compulsory national registries. All individuals with other congenital anomalies than oral clefts or any chronic medical diagnoses by the age of 18 were excluded. A cohort consisting of more than two thousand individuals with isolated cleft and 1.3 million unaffected individuals was followed from the age of 18 through the year 2010.

## Methods

### Study design

**Fig 1:**
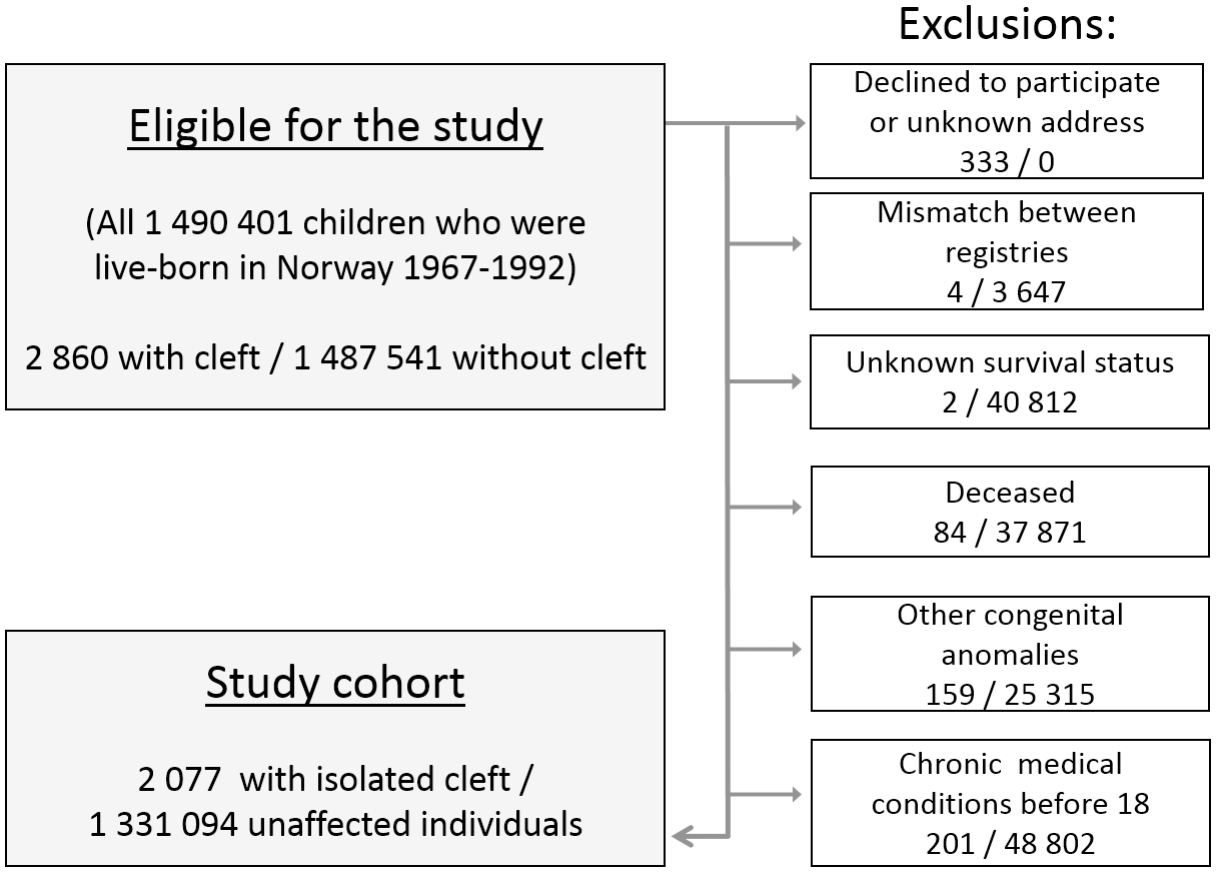
Flowchart describing the selection process of the study population.

All children born in Norway from 1967 to 1992 were considered eligible for the study. This comprised a total of 1 490 401 individuals, including 2 860 in the cleft group (Fig 1). Information was obtained by linking clinical registries of all cleft patients treated in Norway with the following compulsory national registries: The Medical Birth Registry [14], the National Education Database [15], the National Registry [16], the National Insurance Scheme [17] the Norwegian Labor and Welfare Organization [18] and the Norwegian Tax Administration [19]. The unique personal identification number given to all Norwegian citizens simplified the merging procedures.

A total of 166 in the cleft group could not be reached (e.g., unknown address, change of name), and 167 declined participation in the study (Fig 1). Individuals who were not present in all registries or did not reside in Norway in 2010 were excluded. In order to minimize the presence of unobserved disorders we excluded individuals who were registered with any chronic medical condition before the age of 18, or any congenital anomaly other than oral cleft, hydrocele, or hip-dysplasia (Fig 1). The study cohort included a reference group of 1 331 094 unaffected individuals and a group of 2077 individuals with apparently isolated clefts, and was followed from the age of 18 through the year 2010.

The treatment of oral clefts in Norway has since the early 1960’s been coordinated by two regional centers (Haukeland University Hospital, Bergen and Oslo University Hospital, Rikshospitalet, Oslo). The centers have cooperated in the documentation, treatment and follow–up of cleft patients during the whole study period. Experienced clinicians have carried out a thorough and systematic registration of clefts, including comorbidity and classification of cleft morphology. At any given time, treatment at the highest level available has been provided to the patients, free of charge.

The Medical Birth Registry has collected information on all births in Norway since 1967. The registry provided data on birth year, sex, survival status, congenital anomalies, parity, maternal age, and maternal marital status. Congenital anomalies are coded according to the standards of International Classification of Diseases (ICD) [20]. The Medical Birth Registry also provided data on whether an individual became a parent during the study period.

The National Education Database provided information on the education of all individuals in the study and their parents. For parents the following four education levels were used: Master’s degree or PhD (at least five years at university or college); Bachelor’s degree (at least three years at university or college); Completed High School (Intermediate level); Not completed High School (Basic level). Parent’s educational level was recorded when the offspring was 16 years old, and was defined as the level of the parent with the highest education.

The Central Population Registry contained information on parental immigration status (two, one, or no immigrant parents) and marital status of the study participants (married/separated/divorced/widowed vs. single). All residents of Norway are covered by the National Insurance Scheme, which may grant financial support for medical conditions demanding special needs for care or attendance. We collected any such diagnoses that were given before the age of 18.

The Norwegian Labor and Welfare Organization record job related income at an annual basis. Type of occupation was recorded by the Norwegian Tax Administration. Individuals who were employed in 2010 were categorized according to Erikson et al [21], adapted to Norwegian data by Flemmen & Andersen [22]. The categories were *professional*, characterized by responsibility, autonomy or authority (e.g., nurses, lawyers, CEOs, teachers, researchers, and engineers); *manual*, characterized by less responsibility, autonomy and authority, including skilled and unskilled labor (e.g., hairdressers, carpenters, nurse’s aides, cooks, shop assistants, and maids); and *others*, for individuals employed in all other types of occupations (e.g., farmers, lower level store and restaurant managers, nurse’s aides with leadership responsibilities, and the self-employed) [23].

### Statistical analysis

We followed the cohort from the age of 18, and compared each of the groups of isolated CLO, CLP, and CPO with the reference group of unaffected individuals. For most of the analyses we also stratified by sex. The outcome variables were educational achievement at ages 17, 21, and 25, median job related income, job related income in the upper 2006 quartile (yes/no), job related income in the lower 2006 quartile (yes/no), type of occupation, parenting at least one child (yes/no), and marital status (married/not married). Information on all outcome variables except income was available through 2010. The most recent year of recorded income was 2006.

Some individuals had no income in 2006 (mostly young people), and were therefore excluded from the income analysis (n = 229 137). Regarding educational achievement, three educational outcomes were examined: completed basic education by age 17, completed intermediate level education by age 21, and completed higher education by age 25. Basic education consisted of 9–10 years of compulsory school, usually ending the year a pupil turned 16, making this analysis not completely prospective. Intermediate level education required basic education, was optional, and lasted 2–5 years. This was either preparation for skilled labor (carpenter, plumber, hairdresser, cook etc.), preparation for university studies, or both. Higher education consisted of at least three years in college or university (i.e., at least a bachelor’s degree). When considering intermediate and higher education, individuals who had not yet reached the ages of 21 (n = 158 892) and 25 (n = 354 494), respectively, were excluded. Thus, only individuals old enough to have had the possibility of attaining a certain level of education were included in the analyses. Individuals without employment in 2010 (e.g., unemployed, student) were excluded from analyses regarding job type (n = 278 183).

A Poisson regression with robust standard errors [24] was applied when the outcomes were academic achievement, marital status, reproduction, type of occupation, and high/low job related income. This is a numerically robust way to obtain relative risks (RRs) with 95% confidence intervals. Job related income was analyzed using median regression[25], and percent change in median income was reported.

Analyses were adjusted for sex, parity, parental immigration status, marital status of mother at birth (single vs. married/cohabitating), parental level of education when participant was 16 years old, maternal age, and birth year. To account for non-linear effects of age at the end of follow-up, birth year was treated as a categorical variable, with each year being its own category. Analyses were conducted using R version 3.2.3 [26] and Stata 13 [27].

### Ethics

The study was approved by the Regional Committee for Medical and Health Research Ethics. None of the above mentioned registries objected to this approval (the Medical Birth Registry, the National Education Database, the National Registry, the National Insurance Scheme, the Norwegian Labor and Welfare Organization, and the Norwegian Tax Administration). Written consent was provided. Cleft cases born between 1967 and 1992 had all turned 18 in 2010 and letters were sent to each of them, where they were given the opportunity to withdraw from the study. Data were stored on a secure server without information on names or personal identification numbers.

## Results

**Fig 2:**
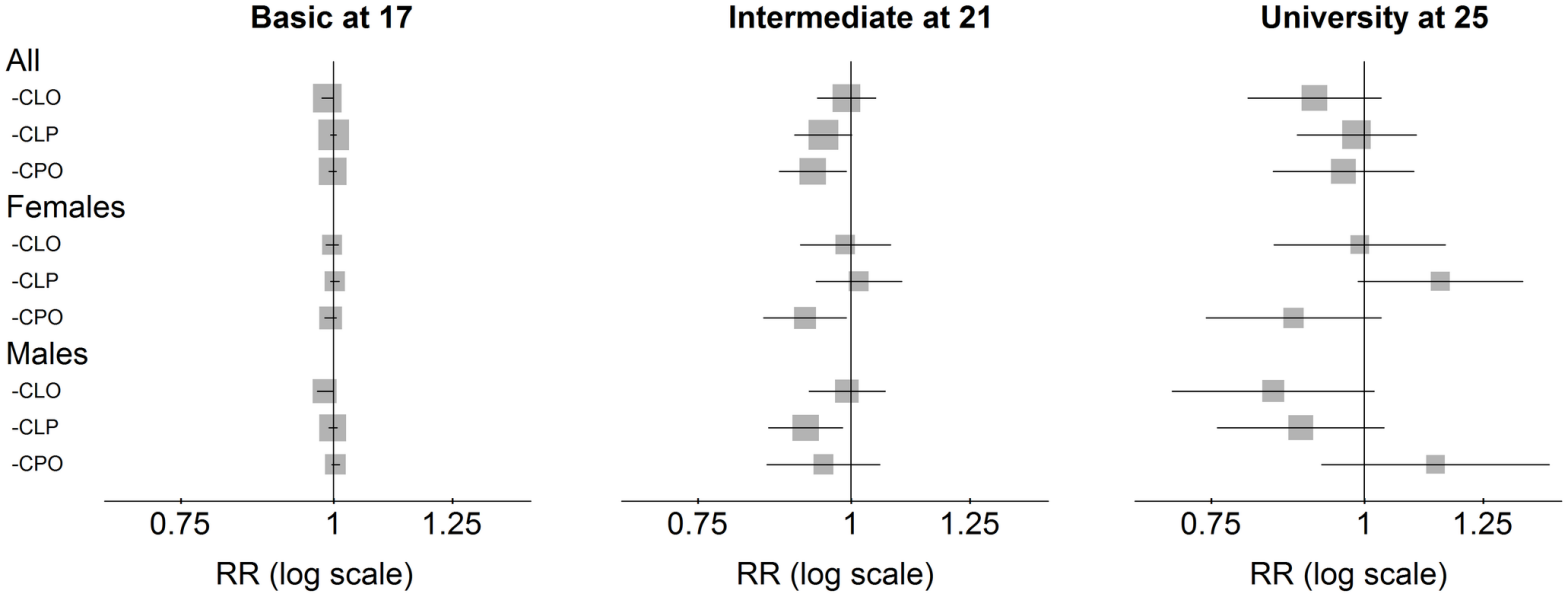
Education attained at ages 17, 21 and 25. Basic: n = 1 333 171 (CLO: 650. CLP: 816. CPO: 611). Intermediate: n = 1 227 480 (CLO: 605. CLP: 763. CPO: 558). University: n = 1 024 482 (CLO: 498. CLP: 659. CPO: 444). All RRs are adjusted for parental education, birth year (categorical), immigration status, maternal marital status.

There were no significant differences in the attainment of basic or high education between the isolated cleft groups and the unaffected reference group (Fig 2). However, marginally reduced proportions in the CLP and CPO groups had completed intermediate education by age 21. In analyses stratified on sex, the completion of intermediate education was reduced among males in the CLP group (RR = 0.92, 95% CI 0.86 to 0.99) and among females in the CPO group (RR = 0.92, 95% CI 0.85 to 0.99).

**Fig 3:**
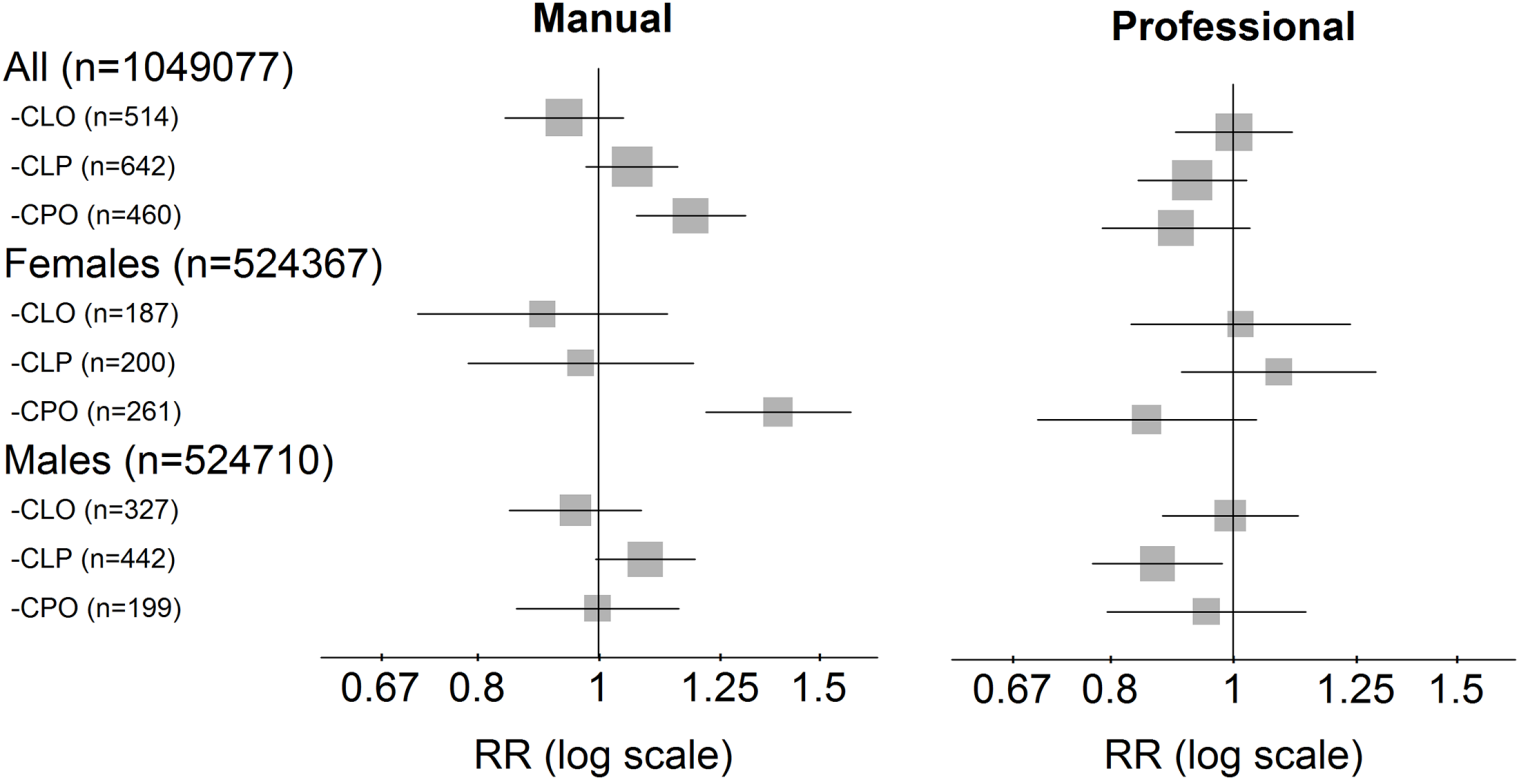
Type of occupation in 2010. Manual: Characterized by less responsibility, autonomy and authority (e.g., hairdressers, carpenters, nurse’s aides, cooks, shop assistants, and maids). Professional: Characterized by more responsibility, autonomy or authority (e.g., nurses, lawyers, CEOs, teachers, researchers, and engineers). All RRs are adjusted for parental education, birth year (categorical), immigration status, and maternal marital status.

About 78.7% of the study cohort was registered with an occupation in 2010 (Table 1). The remaining 21.3% consisted of students, unemployed, retired persons, and people who were too sick to work. Fig 3 shows that the proportion in the manual occupation category was increased for females in the CPO group (RR = 1.39, 95% CI 1.22–1.59), and that males in the CLP group had a decreased probability of employment in the professional category (RR = 0.87, 95% CI 0.78–0.98).

**Table 1:** Characteristics and descriptive statistics for study cohort.

	Reference group†	Any cleft‡	CLO	CLP	CPO
	n = 1331094	n = 2077	n = 650	n = 816	n = 611
Parent 2010, n (%)*					
-Yes	657530 (49.4)	893 (43)	290 (44.6)	343 (42)	260 (42.6)
-No	673564 (50.6)	1184 (57)	360 (55.4)	473 (58)	351 (57.4)
Married 2010, n (%)*					
-Yes	433678 (32.6)	577 (27.8)	187 (28.8)	217 (26.6)	173 (28.3)
-No	897416 (67.4)	1500 (72.2)	463 (71.2)	599 (73.4)	438 (71.7)
Type of occupation 2010, n (%)*					
-Manual	385288 (28.9)	661 (31.8)	188 (28.9)	266 (32.6)	207 (33.9)
-Professional	355688 (26.7)	526 (25.3)	181 (27.8)	217 (26.6)	128 (20.9)
-Other	306485 (23)	429 (20.7)	145 (22.3)	159 (19.5)	125 (20.5)
-Missing	283633 (21.3)	461 (22.2)	136 (20.9)	174 (21.3)	151 (24.7)
Education 2010, n (%)*					
-Basic	456485 (34.3)	650 (31.3)	211 (32.5)	265 (32.5)	174 (28.5)
-Intermediate	563016 (42.3)	889 (42.8)	284 (43.7)	351 (43)	254 (41.6)
-College degree	303112 (22.8)	527 (25.4)	150 (23.1)	195 (23.9)	182 (29.8)
-Missing	8481 (0.6)	11 (0.5)	5 (0.8)	5 (0.6)	1 (0.2)
Sex, n (%)*					
-Males	675674 (50.8)	1253 (60.3)	422 (64.9)	570 (69.9)	261 (42.7)
-Females	655420 (49.2)	824 (39.7)	228 (35.1)	246 (30.1)	350 (57.3)
Parental education, n (%)*					
-Basic	182066 (13.7)	319 (15.4)	81 (12.5)	139 (17)	99 (16.2)
-Intermediate	714449 (53.7)	1105 (53.2)	350 (53.8)	417 (51.1)	338 (55.3)
-Bachelor's degree	316839 (23.8)	471 (22.7)	161 (24.8)	183 (22.4)	127 (20.8)
-Master's degree	116489 (8.8)	180 (8.7)	58 (8.9)	75 (9.2)	47 (7.7)
-Missing	1251 (0)	2 (0)	0 (0)	2 (0)	0 (0)
Immigrant status, n (%)*					
-No immigrant parents	1248048 (93.8)	1966 (94.7)	612 (94.2)	774 (94.9)	580 (94.9)
-One immigrant parent	64972 (4.9)	93 (4.5)	29 (4.5)	38 (4.7)	26 (4.3)
-Two immigrant parents	18074 (1.4)	18 (0.9)	9 (1.4)	4 (0.5)	5 (0.8)
Single mom, n (%)*					
-Yes	141404 (10.6)	231 (11.1)	73 (11.2)	85 (10.4)	73 (11.9)
-No	1189690 (89.4)	1846 (88.9)	577 (88.8)	731 (89.6)	538 (88.1)
Income 2006, n (%)*					
-Yes	1099835 (82.6)	1729 (83.2)	536 (82.5)	699 (85.7)	494 (80.9)
-No	70097 (5.3)	123 (5.9)	44 (6.8)	44 (5.4)	35 (5.7)
-Missing	161162 (12.1)	225 (10.8)	70 (10.8)	73 (8.9)	82 (13.4)
Age in 2010, mean (SD)	31 (7.7)	31 (7.7)	31 (7.7)	31 (7.5)	30 (7.9)

CLO = isolated cleft lip only; CLP = isolated cleft lip with cleft palate; CPO = isolated cleft palate only; SD = standard deviation

*Percentage of top row

^†^Individuals without clefts, any other congenital anomaly except hydrocele or hip dysplasia, or chronic medical conditions.

^‡^Individuals with any cleft, but no other congenital anomalies except hydrocele or hip dysplasia, or chronic medical conditions.

**Fig 4:**
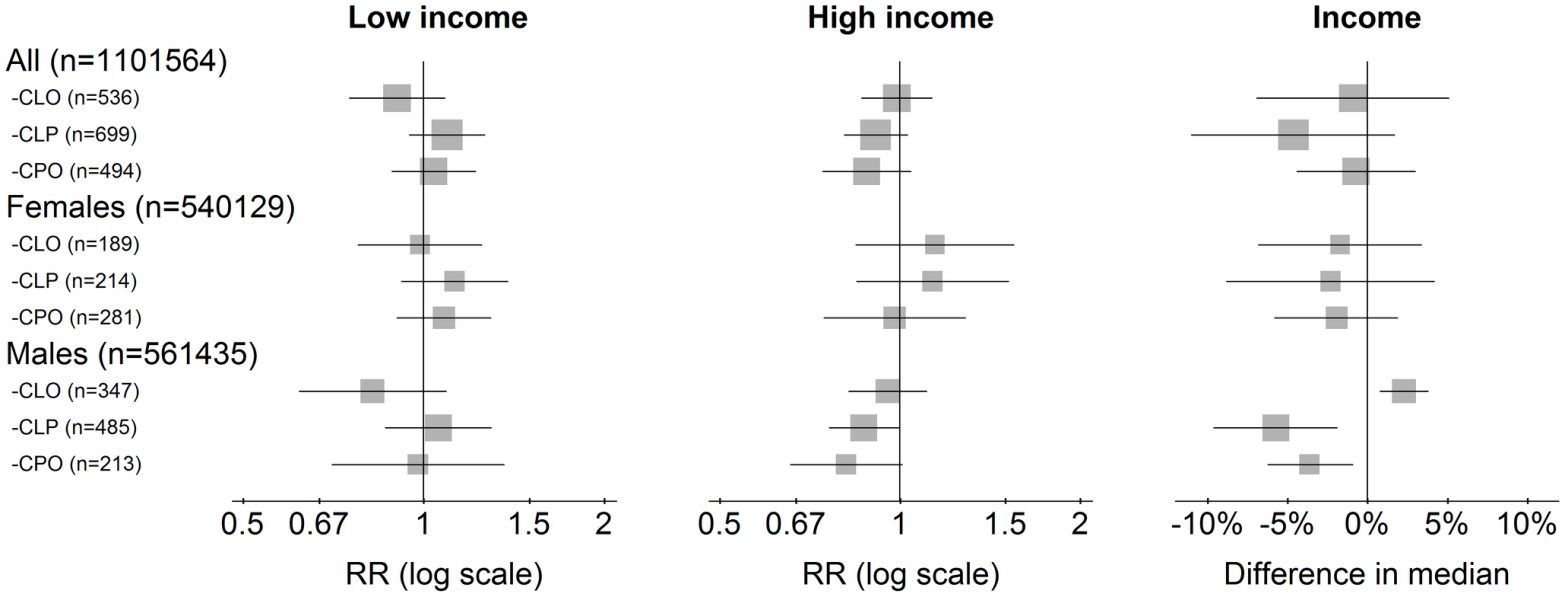
Job related income in 2006. Low income: income in the lower age-specific quartile. High income: income in the upper age-specific quartile. Income: Percent difference in median income between individuals with and without clefts. All RRs and differences in median s are adjusted for parental education, birth year (categorical), immigration status, and maternal marital status.

There were no significant differences between the cleft groups and the reference group regarding their chances of a low or a high income (Fig 4). However, for males there were minor differences in median income. Males in the CLO group had a median income about 2.3 percent *higher* than the males in the reference group (95% CI 0.8 to 3.8), whereas the CLP group (-5.7 percent, 95% CI -9.6 to -1.9) and CPO group (-3.6 percent, 95% CI -6.2 to -0.9) had lower median income.

**Fig 5:**
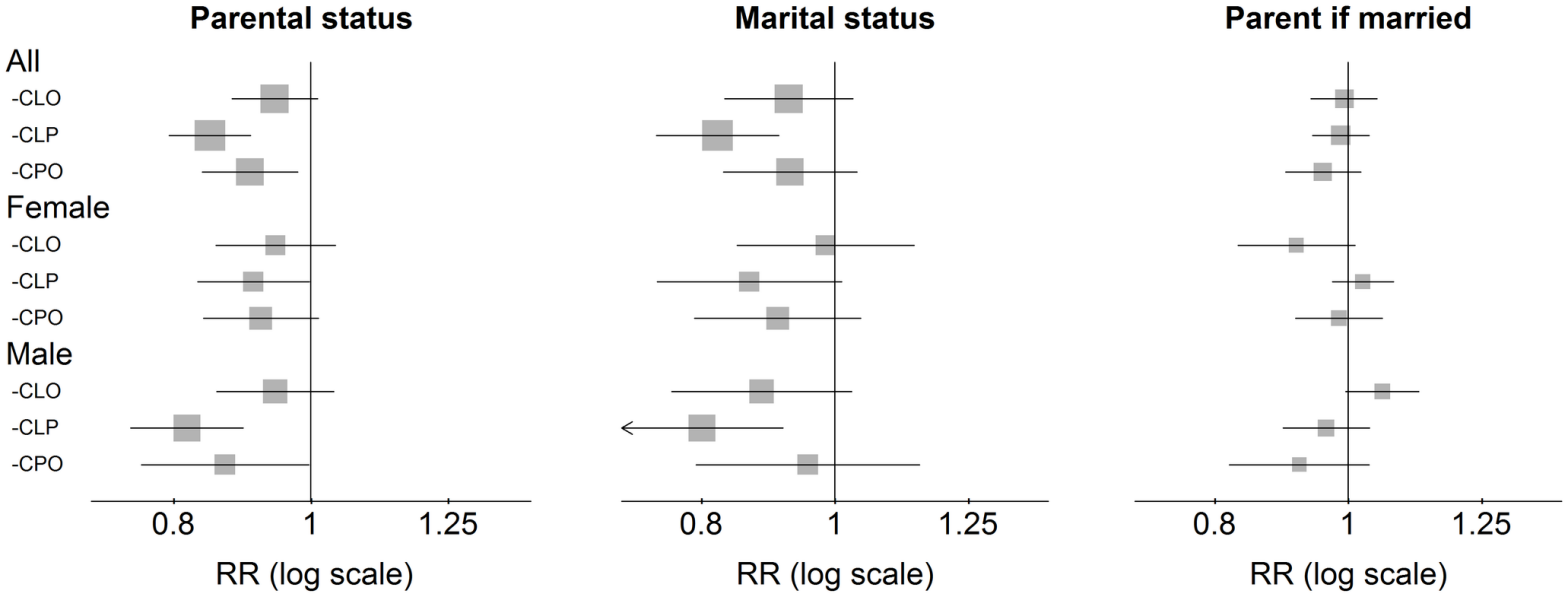
Parental status and marital status. Parental status and marital status: n = 1 333 171 (CLO: 650. CLP: 816. CPO: 611). Parent if married: n = 434 255 (CLO: 187. CLP: 217. CPO: 173). All RRs are adjusted for parental education, birth year (categorical), immigration status, and maternal marital status.

The estimated reproduction rate was slightly lower for the CLP and CPO groups. In the sex stratified analyses, however, a reduction was significant only for males in the CLP group (RR = 0.82, 95% CI 0.75–0.90). A similar pattern was found when considering the chance of getting married (Fig 5). However, among individuals who were married, there was no evidence of a reduced chance of becoming parents in any of the cleft groups.

## Discussion

This national population based cohort study suggests that individuals who were born with an apparently isolated oral cleft have prospects for future socio-economic status, including chances of becoming a parent, that are similar to those in the reference group. For the CLO group, the similarity was remarkable. The CPO group was also similar to the reference group, although males had a slightly lower median income, and women had a somewhat lower chance of completing intermediate education by age 21. Women in the CPO group were also overrepresented in the manual occupation category. The CLP group had some signs of problems, but this seemed to be confined to the males, who were underrepresented in the professional occupation category, had a significantly reduced proportion with intermediate education by age 21, and a lower median income. The chance of marrying or having own children was also lower than in the reference group. These results emerge after extensive attempts to remove cases with other underlying medical problems that sometimes come together with clefts. The good prospects of the cleft groups may be attributed to clinical management and follow-up. For the CLP group, which includes morphologically more severe clefts [28], there were still some apparent challenges, particularly for males.

Studies related to the academic achievement in the cleft groups have often focused on children and adolescents, and have reported ambiguous results. Two large studies with more than 500 cleft cases suggested that school results of children born with clefts were slightly worse than those of unaffected individuals [29, 30]. The first study found that the CPO and CLP groups did worse than the CLO and reference group [29], whereas all groups lagged behind the reference in the second [30]. Both studies accounted for social confounders and to some degree for potential syndromes. A smaller study (n = 256 cleft cases), comparing children born with cleft and their unaffected siblings, reported no differences in academic achievement [31]. Roberts et al., 2012 [10] conducted a relevant meta-analysis of several measures of cognitive functioning and development of individuals born with clefts. When accounting for publication bias and heterogeneity between samples, the only measure where the cleft groups significantly lagged behind unaffected individuals was language development. These studies accounted for the impact of disorders associated with congenital anomalies that were detected at birth, but did not account for disorders that were detected later in childhood. Proper adjustment for social background factors was often lacking and may contribute to differences in results between studies.

Two previous Norwegian studies have shown that males born with clefts are less likely than unaffected individuals to become a parent [32, 33]. Similar results have been found in a study of Danish women [34]. However, neither of these adjusted for social or medical factors. A small questionnaire based study, published by Ramstad et al. in 1995, indicated that Norwegians born with CLP were less likely to marry than unaffected individuals, and when they did marry, they did so at higher age [35]. We confirm that such differences probably exist, but mainly among males. When we included only those who got married, the proportion who became parents was not reduced for any cleft group. This suggests that the reduced fertility was not biological but related to the likelihood of having a partner.

Ramstad et al. [35] also found that married men and single women with CLP had lower income than random controls. Another small questionnaire based study from 1975 (196 cleft cases, 190 unaffected siblings, 209 random controls) found that there were no differences between the cleft group and the controls regarding income and occupational status [36]. Unaffected siblings, however, had a higher income. Both these studies used data that is at least thirty years old today, so it is not obvious that their findings are still relevant.

Educational attainment, reproduction, marital status, and income may affect quality of life, which in turn has been found by several studies to be negatively associated with oral clefting [7, 9]. However, these studies did not account for other chronic medical conditions, outcomes were based on questionnaires as opposed to registry data, and they generally had a low number of participants (n<500) [8]. Because of the differences in methodology and outcomes, the negative association between quality of life and oral clefting cannot be confirmed nor rejected in the current study.

The strengths of this study were the population based design and the large sample size. Information about additional congenital anomalies and chronic medical conditions was available, in addition to several markers of social background. Data was prospectively collected, and loss to follow-up was minimized because the study was based on several compulsory national registries. The clinical registries ensured access to data on all patients treated for clefts in Norway during the study period, except for small portion who declined participation in the study or could not be reached. Proper surgical treatment is important to ensure good results regarding appearance and functioning of individuals born with an oral cleft. The structure of the Norwegian health care system ensures that clefts are detected early, and that high-quality treatment is provided.

One possible limitation of our study was that the proportion of unregistered couples has increased with time, so marital status was probably a better indicator of relationship status for people born early in the study period. We did not have information on parental income or parental type of occupation, and we lacked information on relevant maternal lifestyle factors (e.g., tobacco smoking, alcohol intake, or use of folate supplements). Although we used available information to remove individuals with associated medical disorders, we may still have included some in our cohort. The registration of congenital anomalies in the Medical Birth Registry is not complete (see e.g., Kubon et al. 2007 and Morris et al. 2014 [37, 38]), and we had access to information on chronic medical conditions only for individuals whose parents had requested financial support from the National Insurance Scheme. Failure to exclude such cases should inflate the difference between the cleft groups and the reference group, and could explain some of the marginal differences we find.

Still, an oral cleft did not appear to affect future socio-economic status in Norway. An exception was males with cleft lip and palate, but the differences were small.
